# Theoretical Calculations Facilitating Catalysis for Advanced Lithium-Sulfur Batteries

**DOI:** 10.3390/molecules28217304

**Published:** 2023-10-27

**Authors:** Xue-Ting Fang, Lei Zhou, Chunguang Chen, Dmitri L. Danilov, Fen Qiao, Haitao Li, Peter H. L. Notten

**Affiliations:** 1 School of Physics, Huazhong University of Science and Technology, Wuhan 430074, China; 2 School of Energy and Power Engineering, Jiangsu University, Zhenjiang 212013, China; 3 Department of Chemical Engineering and Chemistry, Eindhoven University of Technology, MB 5600 Eindhoven, The Netherlands; 4 Department of Electrical Engineering, Eindhoven University of Technology, MB 5600 Eindhoven, The Netherlands; 5 State Key Laboratory of Nonlinear Mechanics Institute of Mechanics, Chinese Academy of Sciences, Beijing 100190, China; 6 School of Engineering Sciences, University of Chinese Academy of Sciences, Beijing 100049, China; 7 Institute of Energy and Climate Research Fundamental Electrochemistry (IEK-9), Forschungszentrum Jülich, D-52425 Jülich, Germany; 8 Institute for Energy Research, School of Chemistry and Chemical Engineering, Jiangsu University, Zhenjiang 212013, China; 9 Centre for Clean Energy Technology, University of Technology Sydney, Broadway, Sydney, NSW 2007, Australia

**Keywords:** calculations, catalysis, Lithium-sulfur batteries, polysulfides, conversion kinetics

## Abstract

Lithium-sulfur (Li-S) batteries have emerged as one of the most hopeful alternatives for energy storage systems. However, the commercialization of Li-S batteries is still confronted with enormous hurdles. The poor conductivity of sulfur cathodes induces sluggish redox kinetics. The shuttling of polysulfides incurs the heavy failure of electroactive substances. Tremendous efforts in experiments to seek efficient catalysts have achieved significant success. Unfortunately, the understanding of the underlying catalytic mechanisms is not very detailed due to the complicated multistep conversion reactions in Li-S batteries. In this review, we aim to give valuable insights into the connection between the catalyst activities and the structures based on theoretical calculations, which will lead the catalyst design towards high-performance Li-S batteries. This review first introduces the current advances and issues of Li-S batteries. Then we discuss the electronic structure calculations of catalysts. Besides, the relevant calculations of binding energies and Gibbs free energies are presented. Moreover, we discuss lithium-ion diffusion energy barriers and Li_2_S decomposition energy barriers. Finally, a Conclusions and Outlook section is provided in this review. It is found that calculations facilitate the understanding of the catalytic conversion mechanisms of sulfur species, accelerating the development of advanced catalysts for Li-S batteries.

## 1. Introduction

Energy is of increasingly important concern for global sustainable development since non-renewable fossil fuels are being rapidly depleted. Developing clean and renewable energies, such as wind and solar, is essential to reducing greenhouse gas emissions. Intermittence and fluctuation are crucial challenges when converting these energies to electricity. It is a priority to develop advanced energy storage technologies to utilize wind or solar electricity effectively. Rechargeable batteries provide the invaluable advantage of highly flexible energy storage on various levels [1,2]. Various rechargeable batteries have been developed, including lead–acid, nickel–metal hydride, and lithium-ion batteries [3,4,5]. However, they are plagued by low energy density, which fails to meet the enormous energy storage demands of various application scenarios, like electric vehicles and grids.

Lithium-sulfur (Li-S) batteries have emerged among various advanced battery systems as one of the most promising candidates [6,7,8]. Due to the electrochemical reaction of lithium metal with sulfur by redox processes (2Li + S = Li_2_S), Li-S batteries display a considerably huge energy density of 2600 Wh·kg^−1^, greatly exceeding the current lithium-ion battery systems. Furthermore, they possess the considerable merits of abundant resources, environmental friendliness, and safety. Despite tremendous efforts in exploiting reliable Li-S batteries, their commercialization still has hurdles [9]. The poor conductivity of sulfur cathodes inevitably leads to sluggish electrochemical reaction kinetics with high battery polarization. Moreover, polysulfide intermediates can be dissolved and then diffuse to the electrolyte, resulting in the erosion of lithium anodes. The polysulfide shuttling leads to the heavy failure of electroactive species and poor cycling stability of Li-S batteries. Therefore, it is crucial to synchronously alleviate the polysulfide shuttling and facilitate the electrochemical reaction kinetics, achieving the entire capability of Li-S batteries.

Designing reliable sulfur cathodes is an effective approach to improving the performance of Li-S batteries. Developing advanced sulfur host and separator-modified materials has been demonstrated as a practical approach to promoting cathode conductivity and accelerating sulfur electrochemical kinetics [10,11,12]. Carbons, metals, single atoms, and compounds have been employed as sulfur hosts, which significantly increase the capacity and cycling stability of Li-S batteries resulting from the strong anchoring and catalytic effects on sulfur species [13,14,15,16]. However, it is challenging to discover and screen these host materials by trial and error. Seeking regular theoretical methods for predicting and validating the essential properties of sulfur host materials can facilitate an understanding of electrocatalytic effects during the conversion process in Li-S batteries [17,18]. Theoretical calculations have proven to be a powerful method to examine the electrocatalytic mechanisms in Li-S batteries [19,20,21,22]. Unlike experimental approaches, theoretical calculations can predict the interaction of host materials with sulfur species on the atomic/molecular scale. This considerably facilitates the exploitation of advanced sulfur cathode materials for practical applications.

Theoretical calculations show tremendous advantages in assisting the design and screening of efficient catalyst materials for Li-S batteries. In particular, density functional theory (DFT) calculations have been extensively employed in Li-S batteries. DFT calculations can predict the physical/chemical properties of materials simply using the intrinsic properties of atoms instead of adding any empirical parameters. Currently, DFT is one of the most powerful techniques to simulate the electronic structures of catalyst materials and investigate the interaction between sulfur species and catalyst materials. Based on the DFT calculations, the behavior of catalyst materials can be well explained at the molecular level. The calculated results can further guide the tailoring and optimization of catalyst materials for Li-S batteries. In addition, the Gibbs free energy of the sulfur reduction reaction can be properly obtained with DFT calculations, which can act as an important indicator to evaluate and compare the activities of different catalyst materials. Moreover, the calculation of lithium-ion diffusion barriers and Li_2_S decomposition barriers can provide deep insights into the charge transfer mechanisms in Li-S batteries. All the calculations associated with experimental works accelerate the development of advanced catalyst materials for Li-S batteries.

This review focuses on the theoretical calculations for Li-S batteries, which will help reveal the electrocatalytic mechanisms of the multistep reactions. The first section briefly describes the current advances and issues of Li-S batteries. The electronic structures of catalyst materials are discussed in Section 2 to elucidate their electrocatalytic effects on Li-S batteries. Two important concepts, i.e., binding energy and Gibbs free energy, are presented in Section 3 and Section 4, respectively. Section 5 and Section 6 discuss lithium-ion diffusion energy barriers and Li_2_S decomposition energy barriers. Finally, the Conclusions and Outlook of the review are provided. In this review, we aim to devote our efforts to providing a deep insight into the correlation of the catalyst activities with the structures and the catalytic conversion mechanisms of Li-S batteries with the help of theoretical calculations. The present results will further guide the design and screening of efficient and stable catalyst materials for high-performance Li-S batteries.

## 2. Electronic Structures

The electronic structure of sulfur host materials essentially determines the electrocatalytic activity in Li-S battery conversion reactions. The electronic structure of sulfur host materials can be finetuned with various experimental approaches, such as doping, heterostructures, and defect engineering [23,24,25]. Therefore, it is essential to study the electronic structures of sulfur host materials computationally. This can support the planning of experiments and guide the interpretation of experimental results. DFT calculations are the typical method to study the electronic structures of sulfur hosts, including band structure, density of states, and the charge distribution between sulfur species and sulfur host molecules [26,27,28].

### 2.1. Band Structures

Electronic band structures reveal the electronic levels in crystal structures, which can be used to explain the electronic conductivity of crystals [29,30]. Since sulfur cathodes are electronic conductors, high conductivity benefits the electron transport and the conversion of sulfur species. Therefore, band structure calculations can guide the prediction and screening of efficient sulfur cathode materials. In particular, by calculating the band structures of electrode materials, the width of the band gap can be determined, revealing their conductivity. Materials possessing a negligible energy band gap show metallic properties. However, insulators originating from wide band gaps result in their low conductivity. Due to the relatively narrow band gaps of less than 3 eV, semiconductors display moderate conductivity and can further be enhanced by structure modulation.

**Figure 1:**
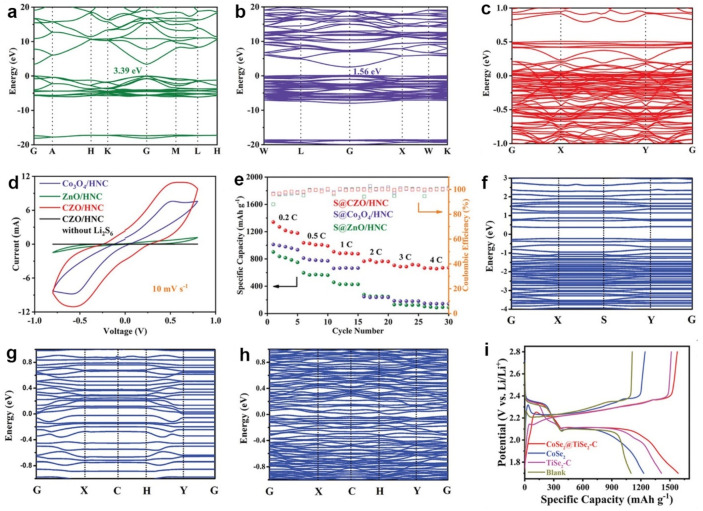
Band structures of (**a**) ZnO, (**b**) Co_3_O_4_, and (**c**) Co_3_O_4_/ZnO. (**d**) CV profiles of symmetric cells. (**e**) Rate capability of different cathodes. (**a**–**e**) Reproduced under the terms of the CC-BY license [31]. Copyright 2023, the authors, published by Wiley-VCH. Band structures of (**f**) CoSe_2_, (**g**) TiSe_2_-C, and (**h**) CoSe_2_@TiSe_2_-C. (**i**) Voltage profiles of different cathodes. (**f**–**i**) Reproduced with permission [32]. Copyright 2023, Wiley-VCH.

DFT calculations have been used to explain the conductivity increase in catalysts by building heterostructures for Li-S batteries [31,32,33,34,35]. Tang and co-workers constructed Co_3_O_4_/ZnO heterojunctions embedded in N-doped carbon nanocages as sulfur hosts (CZO/HNC) [31]. DFT calculations confirmed that Co_3_O_4_/ZnO exhibited an optimized band structure with better conductivity. As shown in Figure 1a–c, ZnO and Co_3_O_4_ possess broad band gaps of 3.39 and 1.56 eV, respectively, implying semiconducting properties. In contrast, the Co_3_O_4_/ZnO heterojunctions revealed a negligible energy band gap, suggesting high conductivity. This result was validated by four-probe resistivity experiments, in which Co_3_O_4_/ZnO heterojunctions displayed the highest conductivity of 6.6 × 10^−3^ S m^−1^. This heterostructure facilitated ion diffusion and promoted the polysulfide conversion with stable Li-S batteries, which was validated by the experimental results. Cyclic voltammetry (CV) of a symmetric cell (Figure 1d) indicated that CZO/HNC exhibited a stronger current response in contrast to Co_3_O_4_/HNC and ZnO/HNC. As a result, sulfur cathodes with CZO/HNC presented the optimized rate capability in comparison with the other two counterparts (Figure 1e).

Recently, another paper on CoSe_2_@TiSe_2_-C heterostructures has also reported the band structures to predict the conductivity (Figure 1f–h) [32]. CoSe_2_@TiSe_2_-C possessed a minor energy band gap of 0.017 eV, which indicated a metallic nature. Relatively broad band gaps were observed in CoSe_2_ (0.589 eV) and TiSe_2_-C (0.024 eV). The highly conductive CoSe_2_@TiSe_2_-C was able to facilitate the conversion kinetics from polysulfides to Li_2_S and promote Li_2_S dissociation. When acting as the interlayer in Li-S batteries, CoSe_2_@TiSe_2_-C allowed the sulfur cathode to deliver the highest capacity (Figure 1i).

### 2.2. Densities of States

Densities of states (DOS) are generally the state number at specific energy levels that electrons can occupy, i.e., the electron state number per unit energy per unit volume. The DOS can be an essential indicator to understand the physical properties of materials since they provide a simple approach to characterizing complex electronic structures. DOS calculations can ascertain the overall state distribution as a function of spacing and energy between energy bands in semiconductors. DOS are typically analyzed from two aspects: the local DOS (LDOS) and the partial (or projected) DOS (PDOS). The LDOS signify that specific atoms of the system contribute electronic states to various parts of the energy spectra. The PDOS indicate the projection of atomic orbitals (s, p, or d) on the densities of states, which provides contributions based on the angular momentum.

The DOS calculations play a critical role in predicting and analyzing the electrochemistry of Li-S batteries. The DOS analyses can readily identify the width of the band gap of electrode materials, which evaluates the conductivity of electrode materials. For instance, electrode materials with band gaps of more than 3 eV between the top of the valence band and the bottom of the conduction band are considered to have insulating properties. A band gap between 1 and 3 eV calculated from DOS indicates that electrode materials are semiconductors. Electrode materials with metallic conductivity present narrow band gaps of less than 1 eV. Therefore, a smaller band gap means a better conductivity for electrode materials. Since the electrochemical conversion of sulfur cathodes is rather sluggish due to the insulating nature of sulfur, catalyst materials with good conductivity are a prerequisite for rapid charge transfer and catalytic conversion of sulfur cathodes [16]. As a result, the electrochemical conversion of elemental sulfur to the discharging product Li_2_S is significantly accelerated owing to the enhanced conductivity of the electrode materials [17]. The detrimental shuttle effect of polysulfides can be further suppressed. Therefore, Li-S batteries are expected to achieve high sulfur utilization and excellent cycling performance.

Moreover, as the strong interaction between catalysts and sulfur species can affect the DOS of catalyst molecules, the detailed DOS analyses interpret the orbital overlapping or hybridization between sulfur species and catalyst molecules, implying the catalytic mechanisms of Li-S batteries. In addition, DOS calculations can also determine the d-band center (ε_d_) of catalysts in Li-S batteries, which is a critical descriptor to analyze the catalytic activities [25,36]. According to the d-band theory, catalysts with a higher value of ε_d_ calculated from DOS suggest a stronger catalytic activity, which better promotes the conversion kinetics for Li-S batteries.

#### 2.2.1. Conductivity Analyses

**Figure 2:**
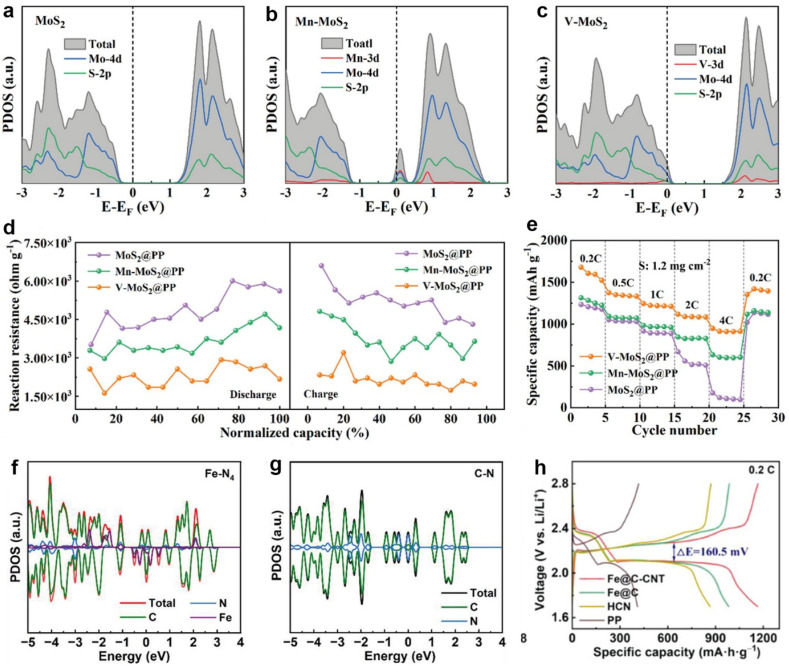
(**a**–**c**) PDOS of MoS_2_, Mn-MoS_2_, and V-MoS_2_, respectively. (**d**) In situ reaction resistance of Li-S batteries. (**e**) Rate capability of different cathodes. Reproduced with permission [41]. Copyright 2023, Wiley-VCH. (**f**,**g**) DOS of Fe-N_4_ and C-N. (**h**) Voltage profiles of sulfur cathodes with different interlayers. Reproduced with permission [43]. Copyright 2023, Wiley-VCH.

Like band structures, DOS can also reveal the conductivity of materials by determining the band gap [37,38,39,40]. Tuning the d-band electronic structures of MoS_2_ by introducing dopants, Liu et al. designed two catalysts, Mn-doped MoS_2_ (Mn-MoS_2_) and V-doped MoS_2_ (V-MoS_2_), to boost the conversion process of sulfur species [41]. As shown in Figure 2a–c, the PDOS of MoS_2_ exhibited the semiconducting property. With Mn doping, Mn-MoS_2_ showed a significant decrease in the band gap and an upshifted Fermi level. A new energy level appeared close to the bottom of the conduction band and went across the Fermi level, suggesting the n-type doping. With V doping, the V-MoS_2_ Fermi level downshifted to the valence band, implying the p-type doping and enhanced conductivity. DOS calculations revealed that cation doping effectively improved the electrocatalytic activities of inactive MoS_2_ towards Li-S batteries. This prediction was consistent with the determined reaction resistances in charging and discharging (Figure 2d). Sulfur cathodes with V-MoS_2_ on the polypropylene separator (V-MoS_2_@PP) show a smaller resistance, implying smaller interfacial reaction barriers during the sulfur conversion process. Therefore, V-MoS_2_@PP enabled sulfur cathodes to achieve the best rate capability (Figure 2e).

With LDOS and PDOS analyses, Chen et al. validated the synergistic adsorption–electrocatalysis of the SnS_2_-MXene Mott–Schottky heterostructures due to the interfacial built-in electric field (BIEF) [42]. The LDOS of SnS_2_ present a band gap of about 2 eV between the conduction band and the valence band, indicating a semiconducting property of SnS_2_. By contrast, the band gap disappeared when SnS_2_ contacted MXene, implying the metallic property of the SnS_2_-MXene heterostructure. Meanwhile, the LDOS indicated that the Ti atom in MXene mainly contributed to the electronic state of the conduction band close to the Fermi level. On the other hand, the S 3p orbitals of SnS_2_ exhibited a band gap, meaning that the S orbital did not contribute to the SnS_2_ conductivity. When forming a BIEF, the state distribution of the S 3p orbitals became broader and delocalized. The band gap disappeared, and the Fermi level passed across the S 3p orbitals, improving conductivity. Similarly, the valence band of Sn 4d orbitals from SnS_2_-MXene upshifted significantly compared to SnS_2_, causing a narrower band gap. As a result, SnS_2_-MXene with metallic conductivity displayed favorable conductivity with rapid charge transfer for the Li-S electrochemistry.

Guo et al. verified that introducing single iron atoms into carbon nanoboxes (Fe@C) enhanced electron transport [43]. From the DOS of Fe-N_4_ and N-doped carbon (C-N) shown in Figure 2f,g, Fe-N_4_ showed more peaks close to the Fermi level than C-N, suggesting that the doped Fe improved the conductivity of C-N. The PDOS of Fe 3d orbitals showed that Fe 3d contributed to the sharp peaks. Thus, Fe 3d was responsible for tailoring the polysulfide anchoring and electron transfer. Benefiting from the structural advantages, the fabricated Fe@C-wrapped carbon nanotube (Fe@C-CNT) interlayer delivered the highest capacity of approximately 1200 mAh g^−1^ with the lowest polarization voltage of 160.5 mV (Figure 2h).

In addition, Wang et al. indicated that the W doping increased the DOS near the Fermi level, resulting in a metallic nature with favorable electron transfer properties for W-doped Co_3_O_4_ [44]. These merits facilitated charge transfer in the redox conversion of polysulfides. Ma et al. designed TiO_2_ anatase/rutile homojunction (A/R-TiO_2_) with the effective catalytic conversion of polysulfides [45]. Based on the DOS calculations, A/R-TiO_2_ possessed excellent conductivity. In contrast to the band gaps from the DOS of A-TiO_2_ and R-TiO_2_, A/R-TiO_2_ exhibited a continuous distribution of electronic states around the Fermi level, causing the metallic nature with improved conductivity.

#### 2.2.2. Interaction between Catalysts and Polysulfides

**Figure 3:**
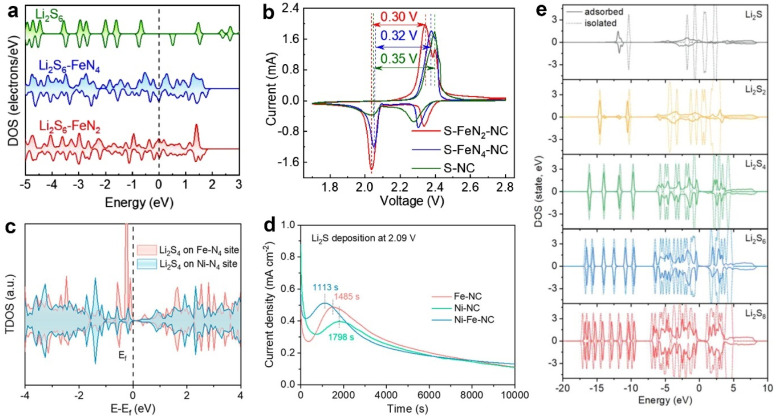
(**a**) The S 2p DOS profiles of Li_2_S_6_, Li_2_S_6_-FeN_4_, and Li_2_S_6_-FeN_2_. (**b**) CV of different cathodes. Reproduced with permission [47]. Copyright 2022, Elsevier. (**c**) TDOS of Ni-N_4_ and Fe-N_4_ sites with Li_2_S_4_ adsorption. (**d**) Li_2_S deposition curves. Reproduced with permission [48]. Copyright 2023, American Chemical Society. (**e**) Comparison of DOS of the isolated and adsorbed Li_2_S*_n_*. Reproduced with permission [40]. Copyright 2023, Wiley-VCH.

The interaction between catalysts and polysulfides can be obtained by analyzing the DOS of isolated and adsorbed polysulfides. To determine the conversion mechanisms from Li_2_S_2_ to Li_2_S in two metal–organic frameworks (ZnCo-MOF and Zn-MOF), Zhu et al. analyzed the orbital overlapping of the catalysts and the S atom of the LiS radical intermediate [46]. The Co d orbitals considerably overlapped with the S p orbitals, forming strong hybrid orbitals between ZnCo-MOF and LiS. By comparison, the S and Zn of Zn-MOF-LiS showed weak orbital hybridization. The DOS calculations revealed a stronger interaction between Co and LiS. Wang et al. confirmed the enhanced polysulfide immobilization by Fe single atoms [47]. A comparatively isolated pattern was revealed from the PDOS of S p orbitals of bare Li_2_S_6_ (Figure 3a). However, after adsorption, Li_2_S_6_-FeN_4_ and Li_2_S_6_-FeN_2_ displayed continuous S p-DOS patterns with a significant distribution near the Fermi level, suggesting the hybridization of S p and the metal d orbitals. This result validated the favorable electronic structure of Fe-N_2_ for enhanced sulfur redox kinetics. The authors fabricated the single-atom Fe on N-doped carbon (FeN_2_-NC) to catalyze sulfur cathodes. From the CV shown in Figure 3b, sulfur cathodes with FeN_2_-NC (S-FeN_2_-NC) present the strongest peak currents with the smallest voltage difference, suggesting mitigated polarization and enhanced reaction kinetics.

Recently, Yang et al. studied the bidirectional electrocatalytic effect of Ni-N_4_ and Fe-N_4_ dual sites co-anchored in carbon nanocages (Ni-Fe-NC). The TDOS calculations in Figure 3c indicate that the Fe-N_4_ center showed a stronger electron density near the Fermi level than the Ni-N_4_ center after the Li_2_S_4_ adsorption, implying that Fe-N_4_ rapidly catalyzed the polysulfide conversion [48]. The Li_2_S deposition measurements shown in Figure 3d testified to the calculations, in which Fe-NC presented the faster Li_2_S nucleation time with a higher deposition capacity of about 195 mAh g^−1^ compared with Ni-NC (147 mAh g^−1^). Dai et al. studied the DOS of Li_2_S*_n_* (1 ≤ *n* ≤ 8) isolated and adsorbed on NiCo_2_S_4−*x*_. As shown in Figure 3e, compared to the isolated Li_2_S*_n_*, the adsorbed Li_2_S*_n_* displayed gradually decreased DOS with the decrease in *n* of Li_2_S*_n_* [40]. The calculations proved the improved anchoring ability of polysulfides by S-vacancy NiCo_2_S_4−*x*_, resulting in enhanced sulfur utilization.

#### 2.2.3. d-Band Center Calculations

**Figure 4:**
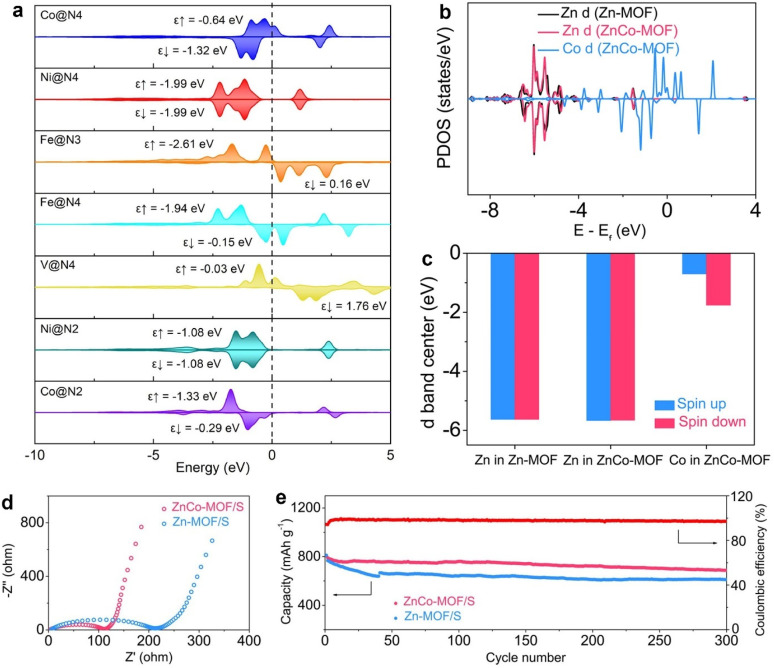
(**a**) The d-band center of various SACs before polysulfide adsorption. Reproduced with permission [49]. Copyright 2022, Elsevier. (**b**) The calculated PDOS and (**c**) ε_d_ of Zn and Co from two MOF catalysts. (**d**) EIS spectra and (**e**) cycling performance of different sulfur cathodes. Reproduced with permission [46]. Copyright 2023, Wiley-VCH.

Summarizing the computational hydrogen electrode (CHE) method, Yi et al. established a theoretical model to uncover the catalytic conversion mechanisms from Li_2_S_2_ to Li_2_S [49]. These predicted mechanisms were determined by modeling Fe, Co, Ni, and V single-atom catalysts (SACs). The PDOS in Figure 4a demonstrates the d-band center (ε_d_) from the investigated SACs before the adsorption. The PDOS of Co, Fe, and V atoms contained two ε_d_ of spin-up (ε↑), and spin-down (ε↓) due to the spin polarization, of which the higher ε_d_ was considered since the d electrons at the higher energy level were more active to interact with coordinated atoms, like S. The ε_d_ of Fe@N_4_ (−0.15 eV) and Fe@N_3_ (0.16 eV) were nearest to the Fermi level among all SACs. The nearly half-occupied d orbitals of the Fe atom allowed moderate adsorption with *LiS, *Li_3_S_2_, and Li_2_S_2_. The highest ε_d_ of 1.76 eV from V@N_4_ suggested that almost the whole d orbitals of V atoms were unoccupied. This indicates that V@N_4_ has the strongest adsorption with the sulfur species having the lone electron pairs. By contrast, Ni@N_4_ and Ni@N_2_ showed more negative ε_d_, meaning the d orbital was nearly entirely occupied. Therefore, the energy level of the d-orbital electrons failed to match the lone electron pairs from sulfur, leading to the weak interaction of sulfur species with the SACs.

Zhu et al. developed a catalyst composed of bimetallic MOF nanoboxes (ZnCo-MOF NBs) to catalyze sulfur cathodes [46]. To unravel the catalytic conversion of sulfur species at the atomic level, the authors calculated the PDOS of two ZnCo-MOF and Zn-MOF catalysts. As shown in Figure 4b, the Co d states of ZnCo-MOF were significantly close to the Fermi level in contrast to the Zn d states, indicating that the Co d orbitals were dominantly active. The Co active center of ZnCo-MOF was thus responsible for the polysulfide adsorption and catalytic conversion. By comparison, the Zn d states of Zn-MOF were basically located in the same position as that of Zn in ZnCo-MOF. Figure 4c presents the calculated ε_d_ of Co and Zn from the two MOF catalysts. The higher Co ε_d_ suggests higher catalytic activities than the Zn sites, according to the d-band theory. Due to the enhanced catalytic effects from ZnCo-MOF, sulfur cathodes with ZnCo-MOF (ZnCo-MOF/S) displayed improved interfacial kinetics from the electrochemical impedance spectroscopy (EIS) spectra (Figure 4d). ZnCo-MOF/S, therefore, achieved a reversible capacity of 688 mAh g^−1^ after 300 cycles at 0.5 C (Figure 4e).

With DOS calculations, the Chen group predicted the ε_d_ of a series of catalysts for sulfur cathodes, which provided a theoretical understanding of the electrochemical conversion mechanisms of sulfur species [47,50,51,52]. By analyzing the DOS of Co_3_O_4_ and Fe-doped Co_3_O_4_ (Fe-Co_3_O_4_), the Chen group observed that Fe-Co_3_O_4_ possessed a higher ε_d_ (−1.9569 eV) than that of Co_3_O_4_ (ε_d_ = −2.0100 eV) [50]. The higher ε_d_ increased the energy of the antibonding orbitals, thus strengthening the chemical interaction of Fe-Co_3_O_4_ with polysulfides. This positive effect considerably benefited the polysulfide anchoring and catalytic conversion in Li-S batteries. In addition, they developed a quasi Zr-based MOF (Q-Zr-BTB) to catalyze Li-S batteries [51]. The deficient Zr-O coordination in Q-Zr-BTB lifted the Zr ε_d_ close to the Fermi level, facilitating the binding of the Zr with polysulfides. Consequently, Q-Zr-BTB enhanced the redox kinetics of sulfur cathodes. Furthermore, they predicted that single-atom Co-B_2_N_2_ sites exhibited a higher ε_d_ than Co-N_4_, offering a stronger interaction of Co with sulfur species [52]. The fabricated sulfur hosts composed of Co-B_2_N_2_ sites anchored on carbon nanotubes effectively catalyzed the polysulfide conversion.

### 2.3. Charge Distribution

Understanding the interaction between molecules is beneficial to obtaining insights into the nature of intermolecular bonding. This understanding effectively guides the design of catalyst materials. Charge distribution calculations are powerful approaches to evaluating intermolecular interactions, which can uncover atoms’ electronic structures and chemical environments. With Bader charge analysis and differences in charge density, the charge transfer and the numerical values for the bond strength of interacting atoms or molecules can be evaluated.

In Li-S batteries, the adsorption and catalysis processes of sulfur species on catalysts involve complicated electron transfers, which are challenging to investigate with experimental approaches. By contrast, charge distribution analyses can clearly present the electron transfer and charge density at the interface of catalysts and sulfur species at the molecular level. Therefore, the chemical bonding interaction between catalyst molecules and sulfur species can be identified, contributing to the understanding of catalytic mechanisms in Li-S batteries.

**Figure 5:**
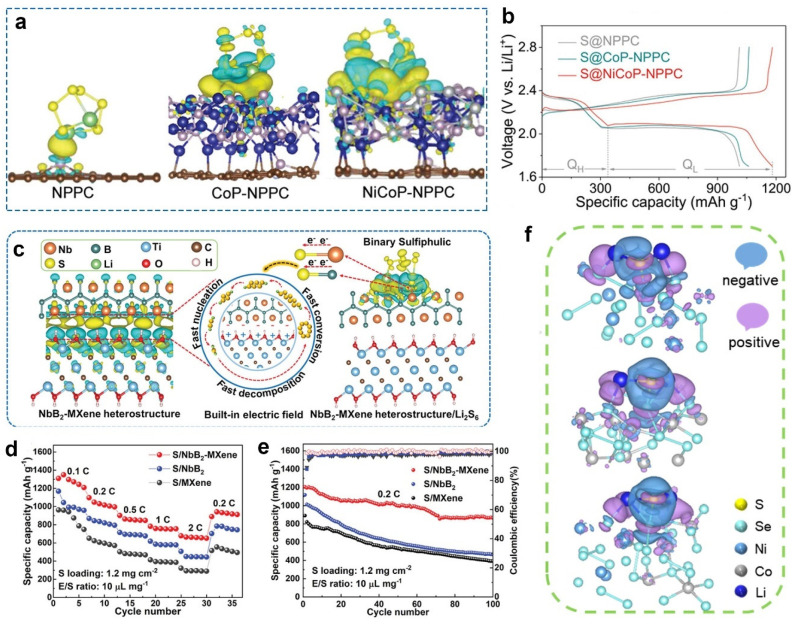
(**a**) Charge density difference for Li_2_S_6_ on NPPC, CoP–NPPC, and NiCoP-NPPC. Yellow and blue suggest the accumulation and depletion of electron charge density, respectively. (**b**) Voltage profiles of different cathodes. Reproduced with permission [53]. Copyright 2023, Wiley-VCH. (**c**) Charge density difference in NbB_2_ and MXene and the adsorption–electrocatalysis mechanism of NbB_2_-MXene heterostructure. (**d**) Rate performance and (**e**) cycling performance of different cathodes. Reproduced with permission [54]. Copyright 2023, Wiley-VCH. (**f**) Charge density difference of NiSe_2_, CoSe_2_, and NiSe_2_-CoSe_2_. Reproduced with permission [38]. Copyright 2023, Elsevier.

Modulating the electronic state of metal phosphides, Zhou et al. incorporated N- and P-doped porous carbons into Ni and Co phosphides nanoparticles (NiCoP-NPPC) as catalysts for Li-S batteries [53]. The tailoring improved the reaction kinetics of sulfur cathodes and achieved a dendrite-free lithium anode. The authors analyzed the interaction between sulfur and transition metals using charge density difference analyses. Figure 5a illustrates that NiCoP-NPPC hybrids with strong Li_2_S_6_ binding energies possessed a localized charge. In contrast to NPPC and CoP-NPPC, NiCoP-NPPC exhibited a higher electron density. Meanwhile, the accumulated and depleted charge might accelerate the charge transport from NPPC to NiCoP. The calculation results indicated a distinct interfacial charge interaction, improving the anchoring and considerably facilitating the electrochemical kinetics of sulfur cathodes. Consequently, sulfur cathodes with CoP-NPPC obtained a high initial capacity of 1184 mAh g^−1^ at 0.5 C (Figure 5b).

By calculating the interfacial charge distribution, Lu et al. confirmed the presence of the built-in electric field (BIEF) in the NbB_2_-MXene heterostructure [54]. Figure 5c shows the accumulated electrons (yellow) at NbB_2_ sites and the gathered holes (blue) at the MXene sites of NbB_2_-MXene heterostructures. Due to the electron flow from MXene to NbB_2_, the NbB_2_-MXene heterostructure ended up with moderate anchoring with polysulfides, accelerating the diffusion of lithium ions and polysulfides. The redistributed charge and the defective boundaries in the heterostructure resulted in more exposed active sites, thus enlarging the anchoring sites and catalytic active sites. These structural advantages enhanced the electrochemical kinetics of the polysulfide conversion. When being used as the sulfur host, NbB_2_-MXene allowed sulfur cathodes to present a boosted rate performance, with a capacity of 679 mAh g^−1^ even at 2 C (Figure 5d). Moreover, the composite cathodes maintained a high capacity of 866 mAh g^−1^ at 0.2 C after 100 cycles (Figure 5e).

The charge density difference analyses can further support the DOS results of the bonding states. Yi et al.’s DOS calculation proved the Fe-S bonding of the adsorbed ^∗^LiS and ^∗^Li_3_S_2_ on the Fe single atoms (* is the active site of catalysts) [49]. The authors observed the clear accumulated charge between Fe and S atoms from the charge density difference. By inducing strain relaxation, Sun et al. tuned the structures of bimetallic MoNi_4_ nanoalloys [55]. The strained MoNi_4_ (s-MoNi_4_) balanced the anchoring and catalytic effects on Li-S batteries. The introduction of the lattice strain altered the bond length of Ni-Mo, broadening the d band and downshifting the d-band center toward the Fermi level. Using the Bader charges, the authors studied the charge transfer interaction of Li_2_S with s-MoNi_4_ and pure MoNi_4_. After adsorbing Li_2_S, s-MoNi_4_ accepted the charge of 0.8227 e, whereas MoNi_4_ accepted 0.9127 e. This result indicated that a slight charge transfer occurred between s-MoNi_4_ and Li_2_S, meaning a weaker charge interaction and thus facilitating the desorption of polysulfides.

Wan et al. analyzed the electronic distribution of the ultra-thin NiSe_2_-CoSe_2_ heterostructured nanosheets using charge density difference calculations [38]. In contrast to NiSe_2_ and CoSe_2_, NiSe_2_-CoSe_2_ displayed a localized enhancement of the positive/negative electron clouds (Figure 5f). It can be concluded that more electron transfers occurred between the interfacial domains of NiSe_2_-CoSe_2_ heterostructures, which significantly anchored and catalyzed sulfur species. With simulated deformation charge density calculations, Dong et al. validated that the dual Fe-Co single-atom pairs exhibited stronger adsorption towards Li_2_S_6_ than Fe or Co single atoms [56]. The dual Fe-Co sites with combined effects induced the charge redistribution of atom pairs, promoting polysulfide reduction and Li_2_S decomposition. Wang et al. indicated an enhanced interaction between polysulfides and Ni_3_B nanoparticles on B-doped graphene (Ni_3_B/BG) [57]. The Bader charge calculations revealed more charge transfer from Li_2_S_4_ and Li_2_S to Ni_3_B/BG (0.813 and 0.592 e) than that to BG (0.592 and 0.092 e), which effectively accelerated their conversion kinetics. Furthermore, highly accumulated charge density was detected at the interface of Ni_3_B and BG. The charge redistribution allowed a smooth charge transport channel, boosting sulfur species’ redox kinetics.

## 3. Binding Energy between Sulfur Species and Catalysts

Since the adsorption of polysulfides is critical to suppressing the polysulfide shuttling, the investigation of the interactions of catalysts with sulfur species is beneficial to unraveling the adsorption mechanisms. Therefore, the binding energy can be a good indicator. Since different crystal surfaces of catalysts may possess different binding energies, the selection of representative surfaces for binding energy calculations is critical. X-ray diffraction characterization can provide reasonable surfaces of catalysts for calculations. In addition, choosing surfaces with different atom ratios is another approach because it can fairly evaluate the contribution of different surface atoms to anchoring sulfur species [58]. Binding energy calculations are now a typical approach to predicting or validating the adsorption ability of catalysts towards sulfur species [59]. The binding energy (*E_b_*) between sulfur species and catalysts is defined as follows:

*E_b_* = *E_t_* − *E_s_* − *E_c_*, (1)

where *E_t_*, *E_s_*, and *E_c_* represent the energies of sulfur species adsorbed on catalysts, sulfur species, and catalysts, respectively. A smaller negative *E_b_* value means a stronger binding ability. In some reports, −*E_b_* is defined to indicate the binding energy, i.e., a higher positive *E_b_* value means a stronger binding ability.

Due to the intrinsic property of catalyst materials, their binding energies towards sulfur species vary. Generally, carbon-based catalysts show a relatively weak binding energy, while metal compounds and single atoms possess stronger adsorption towards sulfur species. For instance, Pu et al. compared the binding energies of Fe_3_P and pure carbon towards various sulfur species [60]. Fe_3_P displayed relatively high binding energies in a range of 0.55 to 2.95 eV towards sulfur species, which were stronger than those of pure carbon (0.43–0.65 eV). Due to the electronegativity difference, the sulfur from polysulfides readily interacted with the iron from Fe_3_P. The proper anchoring was beneficial to anchoring sulfur species and accelerating their catalytic conversion. Wang et al. calculated the binding energies of CoP towards sulfur species [61]. The results show that CoP (211) had an excellent adsorption capacity of 2–8 eV towards sulfur species. The similar binding energy of CoP towards sulfur species was also confirmed by Zhang et al. [62]. Structure regulation, like doping, can improve the binding energy of carbon-based catalyst materials [63,64]. Yang et al. validated that introducing pyridinic-N to porous carbon fibers resulted in a strong adsorption energy of −2.20 eV towards Li_2_S_4_ [65].

**Figure 6:**
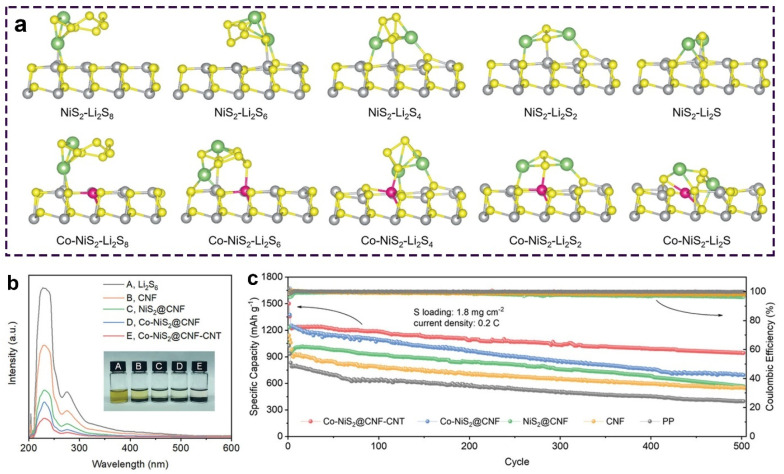
(**a**) Optimized geometries of Li_2_S_8_, Li_2_S_6_, Li_2_S_4_, Li_2_S_2_, and Li_2_S on the (002) surface of NiS_2_ and Co-NiS_2_. (**b**) UV-Vis spectra of the Li_2_S_6_ solution. Insets display digital pictures of the Li_2_S_6_ adsorption experiments. (**c**) Cycle stability of sulfur cathodes with different interlayers. Reproduced with permission [39]. Copyright 2023, Wiley-VCH.

Metal sulfide catalysts display favorable adsorption and catalytic activities towards sulfur species [66,67]. Liu et al. revealed that the cation doping of MoS_2_ significantly promoted the anchoring and the catalytic effects on polysulfides compared to the inactive MoS_2_ [41]. V-doped MoS_2_ possessed stronger adsorption of polysulfides and allowed sulfur cathodes to have a high initial capacity of 1607 mAh g^−1^ at 0.2 C. Dai et al. validated that the Co-doped NiS_2_ (Co-NiS_2_) nanoparticles showed a stronger adsorption towards sulfur species than NiS_2_ [39]. Figure 6a displays the optimum structures of the anchored sulfur species on the NiS_2_ and Co-NiS_2_ (002) surfaces. Polysulfide’s calculated adsorption energies on NiS_2_ were in the range of −0.96 to −2.61 eV. After the Co doping, the calculated values decreased to in the range of −1.36 to −4.16 eV. The calculated results were also validated by the Li_2_S_6_ adsorption measurements with the UV-Vis spectra. Li_2_S_6_ solutions with the Co-NiS_2_ component were nearly transparent after 6 h of adsorption (Figure 6b). Meanwhile, the UV-Vis spectra indicated a decreased intensity resulting from the introduction of Co-NiS_2_, which is consistent with the adsorption experiments. The enhanced adsorption capability of Co-NiS_2_ towards polysulfides benefited the inhibition of the shuttle effects. Accordingly, sulfur cathodes with Co-NiS_2_ catalysts exhibited a highly reversible capacity of 944 mAh g^−1^ at 0.2 C after 500 cycles (Figure 6c).

A single component generally exhibits relatively weak adsorption towards sulfur species. Combining two or more components to construct heterostructures can overcome this shortage [66,68,69]. For example, Zeng et al. designed the ternary heterostructure Na_0.67_Ni_0.25_Mn_0.75_O_2_ (NNMO)-MnS_2_-Ni_3_S_4_ with three active centers to obtain cascade catalysis for polysulfides [70]. Based on the binding energy calculations, NNMO delivered higher binding energies for all sulfur species than MnS_2_ and Ni_3_S_4_. The binding energies of long-chain polysulfides on MnS_2_ were stronger than those on Ni_3_S_4_, while short-chain polysulfides were favorably anchored on Ni_3_S_4_ compared to MnS_2_. Finally, sulfur cathodes with the ternary heterostructure exhibited excellent rate performance. Wang et al. calculated the binding energy of Co_3_O_4_/ZnO heterostructures towards sulfur species [31]. The results suggested that Co_3_O_4_/ZnO displayed the strongest adsorption compared to the individual Co_3_O_4_ or ZnO. A similar conclusion has also been made by Wan et al., who confirmed that the NiSe_2_-CoSe_2_ heterostructure displayed enhanced adsorption of sulfur species compared to NiSe_2_ and CoSe_2_ [38].

**Figure 7:**
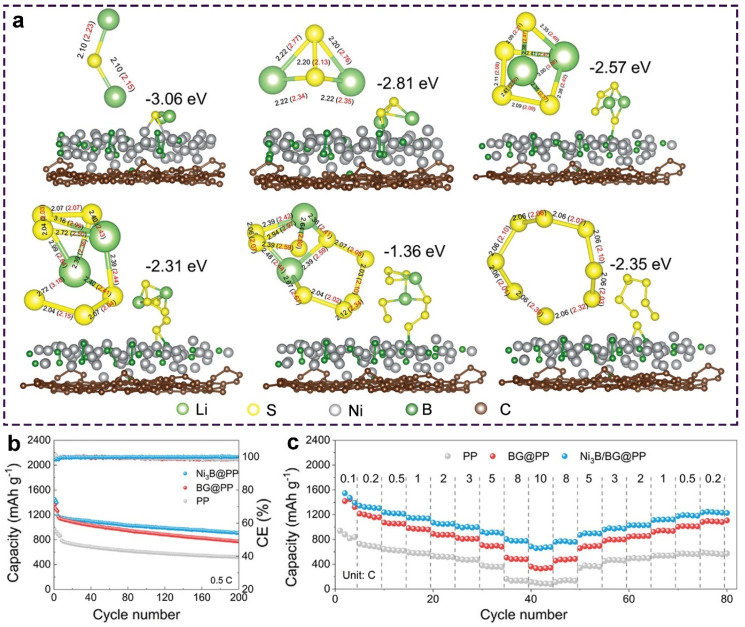
(**a**) Optimum structures of sulfur species adsorbed on Ni_3_B/BG, where the unit of bond length is Å. The values in red indicate the bond length after adsorption. (**b**) Cycling stability at 0.5 C and (**c**) rate capability of different cathodes. Reproduced with permission [57]. Copyright 2023, Wiley-VCH.

Other metal compounds, like oxides and nitrides [71,72,73], have also shown strong adsorption towards sulfur species, which can effectively mitigate the shuttle effects. For example, Wang et al. designed a core-shelled heterostructure containing Ni_3_B nanoparticles dispersed on B-doped graphene (Ni_3_B/BG) to boost the reaction kinetics of Li-S batteries [57]. DFT calculations have determined the adsorption properties of polysulfides. Figure 7a shows various sulfur species’ adsorption energies with optimum structures on Ni_3_B/BG and BG. Due to the binary interaction, the S atoms of polysulfides were significantly bound with B and Ni. That was in accordance with the XPS results and the adsorption experiment. Ni_3_B revealed a stronger anchoring ability to polysulfides compared to BG. Polysulfides adsorbed on Ni_3_B lengthened the bond. This chemical interaction further caused the change in the other bond length. The simultaneous elongation of the S-S and Li-S bonds facilitated the conversion of polysulfides and the Li_2_S deposition process. Ni_3_B/BG exhibited superior adsorption towards polysulfides and desirable catalytic effects, enabling sulfur cathodes to have excellent cycling stability. Sulfur cathodes with Ni_3_B/BG@PP-modified separators maintained a high reversible capacity of 908 mAh g^−1^ at 0.5 C after 200 cycles (Figure 7b). In addition, Ni_3_B/BG@PP allowed sulfur cathodes to obtain a high capacity of 650 mAh g^−1^ even at 10 C (Figure 7c).

## 4. Gibbs Free Energy

Gibbs free energy, denoted as *G*, is defined as follows:

*G* = *H* − *TS*, (2)

where *H*, *T*, and *S* are the enthalpy, temperature, and entropy, respectively. The change in Gibbs free energy (Δ*G*) can indicate the chemical reaction direction under constant pressure and temperature. For example, when Δ*G* shows a positive value, the reaction cannot be spontaneous. Negative values correspond to spontaneous reactions. Since a catalytic reaction involves the adsorption and desorption of reactants on the catalyst’s surface, the Δ*G* calculations can evaluate the activities of catalysts and the rate-determining step. By calculating the correlation between overpotential and Δ*G* in the oxygen reduction reaction, Nørskov et al. successfully explained the activities of metal catalysts [74]. Δ*G* can be an effective descriptor for seeking idealized catalyst materials [64]. Similarly, the Δ*G* calculations have been applied to investigate the electrochemistry of Li-S batteries. For example, Δ*G* can reveal which reaction step is spontaneous and which reaction step is rate-determining in Li-S batteries [75].

Ji and co-workers initially employed the Δ*G* calculation to explain the catalytic activities of single-atom Co for sulfur cathodes [76]. Typically, the electrocatalytic sulfur reduction reaction from S_8_ to Li_2_S during discharging can be considered as follows:

*S_8_ + 2*Li*^+^ + 2*e*^−^ → *Li_2_*S*_8_, (3)

3*Li_2_S_8_ + 2*Li*^+^ + 2*e*^−^ → 4*Li_2_*S_6_*, (4)

2*Li_2_*S*_6_ + 2*Li*^+^ + 2*e*^−^ → 3*Li_2_S_4_, (5)

*Li_2_*S*_4_ + 2*Li*^+^ + 2*e*^−^ → 2*Li_2_*S*_2_, (6)

*Li_2_*S*_2_ + 2*Li*^+^ + 2*e*^−^ → 2*Li_2_S, (7)

where * is the active site of catalysts. The calculated Δ*G* for the corresponding reaction process can be written as follows:

Δ*G*_1_ = *G*(*Li_2_*S*_8_) − *G*(*S_8_) − 2*G*(*Li*), (8)

Δ*G*_2_ = 4*G*(*Li_2_*S*_6_) − 3*G*(*Li_2_*S*_8_) − 2*G*(*Li*), (9)

Δ*G*_3_ = 3*G*(*Li_2_*S*_4_) − 2*G*(*Li_2_*S*_6_) − 2*G*(*Li*), (10)

Δ*G*_4_ = 2*G*(*Li_2_S_2_) − *G*(*Li_2_*S*_4_) − 2*G*(*Li*), (11)

Δ*G*_5_ = 2*G*(*LI_2_*S*) − *G*(*LI_2_S_2_) − 2*G*(*LI*). (12)

In Equations (3)–(7), *G*(*Li*^+^) + *G*(*e*^−^) are written in the form of *G*(*Li*), which is considered in the computational hydrogen electrode approach [74]. Therefore, during the sulfur reduction reaction (SRR) process, the rate-determining step can be determined by the calculated Δ*G.* Du et al. calculated the Δ*G* for two SRR catalysts: N-doped graphene (N/G) and single-atom Co in N-doped graphene (Co-N/G). They validated that the S_8_ reduction to Li_2_S_8_ was an exothermic spontaneous reaction in which Δ*G* < 0. The following reduction processes to discharge products are endothermic or almost thermoneutral. The rate-determining step was the conversion from Li_2_S_2_ to Li_2_S, which presented the highest Δ*G.* Co-N/G indicated a lower Δ*G* than N/G for the Li_2_S_2_ reduction, implying a more favorable reaction pathway. Based on the Δ*G* calculations, various catalyst materials have been predicted, such as single atoms [77], metal oxides [78,79,80], sulfides [81], nitrides [82,83], and heterostructures [84,85], which present accelerated conversion kinetics for Li-S batteries.

Because of the maximized atomic utilization and excellent catalytic activities, single atoms present accelerated conversion kinetics for sulfur cathodes. Single atoms with Fe [48], Co [86], Ni [87], Cu [88], and W [44] as active sites have been confirmed to be promising electrocatalysts, which considerably restrain the polysulfide shuttling. The coordination environment of single atoms plays a key role in catalytic activities. Zhang et al. noticed the catalytic activities of edge-distributed single-atom sites. To achieve edge-distributed single-atom Fe, they incorporated Fe single atoms in N-doped porous carbon (Fe-NPC) into CNTs [89]. This composition promoted polysulfide anchoring and conversion. The Δ*G* calculations supported the favorable catalytic activities resulting from the Fe-N_4_ moieties with edge distribution. In the rate-determining step, Fe-N_4_ with edge distribution decreased the Li_2_S deposition barrier (0.72 eV) compared to the in-plane Fe-N_4_ (0.87 eV), revealing faster redox kinetics. Meanwhile, the energy barrier of the Li_2_S_4_ reduction on Fe-N_4_ with edge distribution also decreased to 0.53 eV in contrast to that on the in-plane Fe-N_4_ surface (0.60 eV), implying promoted polysulfide conversion and hence a mitigated shuttle effect.

Recently, Ren et al. designed a single-atom Fe catalyst containing an S-doped periphery (Fe-NSC), which presented enhanced polysulfide adsorption and facilitated sulfur conversion [16]. Compared with the pristine Fe-N_4_ moieties, the Fe-NSC configuration had more accumulated charge density. The calculations indicated that the Fe-NSC-based catalysts decreased the Δ*G* of the Li_2_S deposition. This result showed a more favorable pathway for sulfur reduction at the Fe-NSC sites, thus achieving excellent cycling life of sulfur cathodes. By regulating the coordination numbers of active sites, the intrinsic catalytic activities of single atoms can be significantly enhanced. Xiao et al. prepared novel single-atom catalysts composed of Fe-N_5_ moieties embedded in the N-doped carbon matrix (Fe-N_5_/NC) [90]. The resultant Fe-N_5_/NC exhibited strong adsorption to polysulfides and considerable catalytic effects on the redox conversion of Li-S batteries. Sulfur cathodes with the Fe-N_5_/NC catalysts displayed a high initial capacity at 0.1 C (1519 mAh g^−1^). The Δ*G* calculations implied that the biggest barrier was the Li_2_S_2_ reduction.

**Figure 8:**
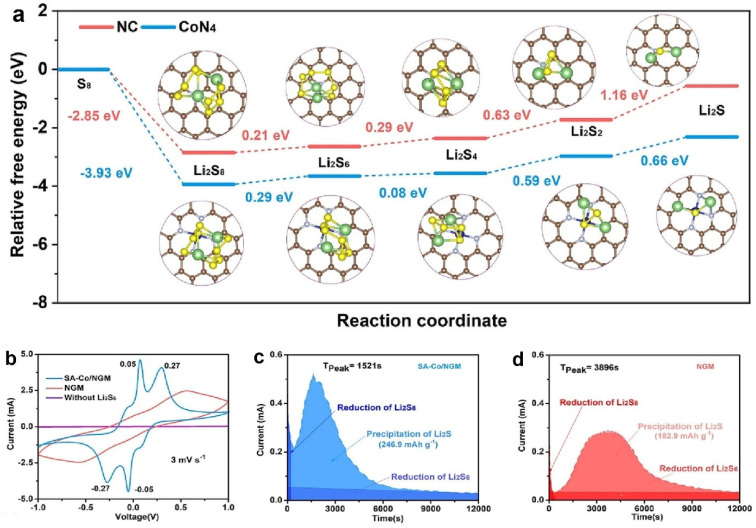
(**a**) Relative free energies from S_8_ to Li_2_S during discharging. Insets show the optimum structures of the sulfur species on NC and CoN_4_. (**b**) CV of Li_2_S_6_ symmetric cells. Li_2_S deposition of (**c**) SA-Co/NGM and (**d**) NGM. Reproduced with permission [93]. Copyright 2023, Elsevier.

Co single atoms have been determined to catalyze the redox conversion of sulfur cathodes [91,92]. Wang et al. engineered planar Co-N_4_ in N-doped graphene mesh. The fabricated single-atom catalysts (SA-Co/NGM) obtained high atom utilization when catalyzing the polysulfide conversion [93]. The authors calculated the Δ*G* of S_8_ to Li_2_S on N-doped carbon (NC) and CoN_4_ to reveal the improved conversion kinetics. Figure 8a shows the optimum structures of the sulfur species with the corresponding Δ*G*. The initial conversion from solid S_8_ to Li_2_S_8_ was the spontaneous exothermic reaction with a negative Δ*G*. The following reduction steps from Li_2_S_8_ to Li_2_S indicated a positive Δ*G*, meaning the endothermic reactions. The reduction from Li_2_S_2_ to Li_2_S with the highest Δ*G* was the rate-determining step, in which CoN_4_ indicated a smaller Δ*G* (0.66 eV) in contrast to NC (1.16 eV). The calculation suggested that the Li_2_S deposition process was thermodynamically more favorable on the CoN_4_ substrate. The facilitated redox kinetics of sulfur cathodes resulting from Co-N_4_ was experimentally validated with CV based on Li_2_S_6_ symmetric cells and Li_2_S deposition. Figure 8b shows that SA-Co/NGM presents two pairs of clear redox peaks with stronger currents than NGM, meaning the accelerated conversion kinetics of sulfur species. Meanwhile, SA-Co/NGM shows a more rapid nucleation time and a larger deposition capacity than NGM (Figure 8c,d).

**Figure 9:**
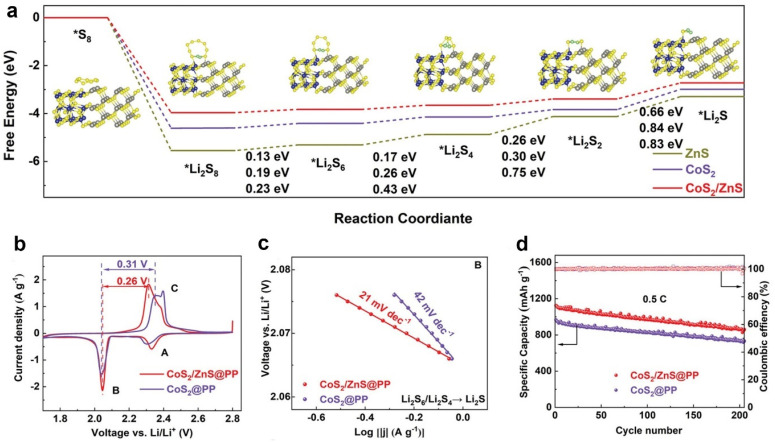
(**a**) Relative free energy from S_8_ to Li_2_S during discharging. Insets show the optimized structures of sulfur species. (**b**) First CV curves and (**c**) Tafel plots calculated from Peak B. (**d**) Cycling performance of different cathodes. Reproduced with permission [33]. Copyright 2023, Wiley-VCH.

Heterostructures integrate the advantages of the individual component to achieve increased catalytic effects on sulfur cathodes. For instance, Meng and co-workers developed a CoS_2_/ZnS heterostructure that can bidirectionally catalyze sulfur cathodes [33]. As shown in Figure 9a, the rate-determining step was considered to be the Li_2_S_2_ reduction to Li_2_S during discharging due to the highest Δ*G.* CoS_2_/ZnS displayed a lower Δ*G* (0.66 eV) than CoS_2_ and ZnS, which indicated the thermodynamically favorable Li_2_S deposition by CoS_2_/ZnS. Accordingly, the facilitated process was confirmed with CV and Tafel analyses. Sulfur cathodes with CoS_2_/ZnS-modified PP separators (CoS_2_/ZnS@PP) displayed a lower voltage difference between the cathodic Peak B and anodic Peak C than that with CoS_2_@PP (Figure 9b). This result indicated that CoS_2_/ZnS decreased the polarization effect of sulfur cathodes. Furthermore, the Tafel slope calculated from Peak B in Figure 9c suggested that CoS_2_/ZnS mitigated the overpotential of the Li_2_S deposition process due to the smaller Tafel slope. Benefiting from the merits of heterostructure, CoS_2_/ZnS@PP achieved prolonged cycling stability for 200 cycles (Figure 9d).

Zhu et al. synthesized heterogeneous MnO-Mo_2_C nanoparticles on porous carbon (MnO-Mo_2_C/C) to host sulfur [94]. MnO-Mo_2_C decreased the energy barrier of Li_2_S deposition to 3.38 eV compared to the single MnO (4.63 eV). Similar conclusions were made by Huang et al., who fabricated La_2_O_3_-MXene heterostructures to promote the conversion kinetics of sulfur cathodes [95].

## 5. Lithium-Ion Diffusion Energy Barriers

Lithium-ion diffusion can be used to evaluate the activities of catalysts for Li-S batteries. CV measurements are typically used to determine the lithium-ion diffusion coefficient [96]. In addition, the diffusion energy barriers of lithium ions on the surfaces of catalysts are good indicators to predict the electrochemical kinetics of Li-S batteries. Cui and co-workers investigated the lithium-ion diffusion on graphene and various sulfides using the climbing-image nudged elastic band method [97]. The calculation showed that the diffusion barriers of sulfides were smaller than those of graphene, which was in accordance with the experimental analyses. A smaller barrier increases the diffusion rate, which benefits the reaction kinetics between lithium and sulfur.

**Figure 10:**
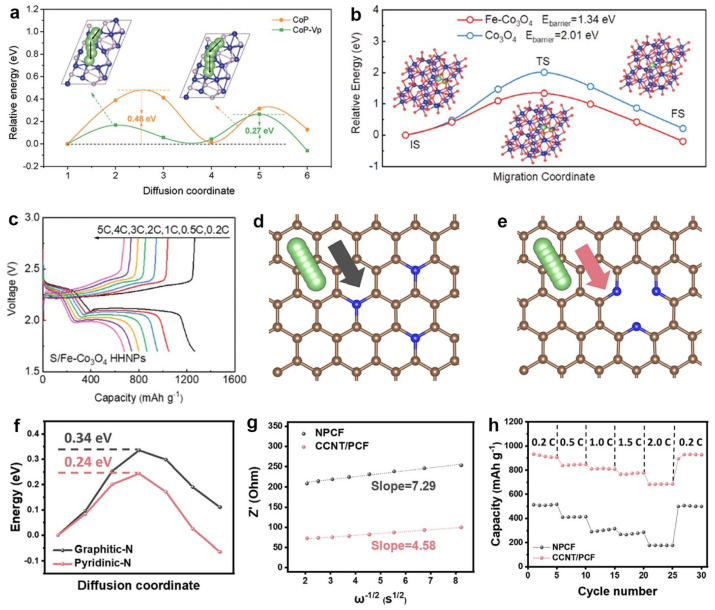
(**a**) The relative energy of lithium-ion diffusion on CoP and CoP-Vp. Reproduced with permission [98]. Copyright 2022, Wiley-VCH. (**b**) Li^+^ diffusion on Fe-Co_3_O_4_ and Co_3_O_4_ and the corresponding geometrical structures. (**c**) Voltage profiles of sulfur cathodes at various C rates. Reproduced with permission [50]. Copyright 2023, Wiley-VCH. Li-ion diffusion pathways in (**d**) graphitic-N and (**e**) pyridinic-N. (**f**) Calculated energy barriers of the lithium-ion diffusion. (**g**) Slope fitting showing the relation between ω^−1/2^ and Z′ from EIS. (**h**) Rate capability of different cathodes. Reproduced with permission [65]. Copyright 2023, Elsevier.

Lithium-ion diffusion can be significantly enhanced by controlling the electronic structures of catalysts. For example, Zhang and co-workers calculated the diffusion barriers of lithium ions on MoS_2_, Mn-doped MoS_2_, and V-doped MoS_2_ [41]. Compared with MoS_2_, the doped MoS_2_ showed smaller diffusion barriers (MoS_2_, Mn-doped MoS_2_, and V-doped MoS_2_ were 0.08, 0.05, and 0.05 eV), suggesting accelerated lithium-ion migration after the introduction of doped elements. This was advantageous to promoting the rate performance of sulfur cathodes. Sun et al. fabricated P-vacancy CoP (CoP-Vp) to promote the polysulfide conversion [98]. Figure 10a shows the lithium-ion diffusion pathways and the corresponding free energy. The lithium-ion diffusion on CoP and CoP-Vp followed a polyline process with double peaks. Two transition states existed between the initial and final stable states. The lower energy barrier of CoP-Vp (0.27 eV) indicated superior lithium-ion diffusion properties, thus improving the rapid conversions of polysulfides. Sulfur cathodes with CoP-Vp showed favorable rate capability at 3 C with a capacity of 738 mAh g^−1^.

Incorporating doped Fe to Co_3_O_4_ nanosheets, Liu et al. suggested that Fe-doped Co_3_O_4_ (Fe-Co_3_O_4_) resulted in numerous active sites which lowered the barrier of the polysulfide conversion [50]. Figure 10b illustrates the diffusion pathways of lithium ions on Co_3_O_4_ and Fe-Co_3_O_4_. The diffusion followed the arc curves, in which the initial state (IS) converted to the transition state (TS) and turned to the final state (FS). Fe-Co_3_O_4_ decreased the diffusion barrier from 2.01 to 1.34 eV, enabling rapid lithium-ion transport for the electrochemical reactions of sulfur cathodes. This merit enabled the sulfur cathode to have a favorable rate capability. Figure 10c shows that sulfur cathodes with Fe-Co_3_O_4_ maintained the two discharge plateaus well, even at 5 C. The considerable decrease in polarization by Fe-Co_3_O_4_ resulted in higher capacities in various current densities.

The surface structures of catalysts significantly influence the diffusion characteristics of lithium ions. Yang et al. employed Ni precursors and CO_2_ conversion to fabricate entangled CNTs on porous carbon nanofiber (PCF) [65]. This method converted the central graphitic-N in the graphene plane to the edge-site pyridinic-N and pyrrolic-N, which boosted the adsorption towards sulfur species. The resulting CO_2_-derived CNTs on PCF (CCNT/PCF) exhibited better catalytic activities than the non-porous carbon nanofiber (NPCF). Figure 10d,e show the lithium-ion diffusion pathways on the graphitic-N- and pyridinic-N-doped carbon, respectively. The lithium-ion diffusion barrier along pyridinic-N was calculated to be 0.24 eV (Figure 10f), which was lower than that along graphitic-N (0.34 eV). This result indicated that pyridinic-N facilitated the lithium-ion diffusion kinetics and improved Li-S batteries’ performance. The Li-ion diffusion was further experimentally determined using EIS analyses. As shown in Figure 10g, by fitting the linear relationship between ω^−1/2^ and Z′, CCNT/PCF exhibited a smaller slope than NPCF, meaning a faster lithium-ion diffusion behavior. Therefore, CCNT/PCF achieved significantly larger capacities than NPCF at various current densities (Figure 10h).

Sun et al. confirmed that incorporating high oxygen contents into the CoP surface accelerated the electrochemical kinetics of polysulfides [99]. The authors calculated the diffusion of lithium ions on CoP with low and high oxygen contents. Lithium ions diffused on CoP with a high oxygen content underwent a decreased barrier of 0.47 eV, meaning a rapid lithium-ion diffusion and accelerated electrochemical conversion of polysulfides. A strain relaxation method has also been reported to tailor the anchoring and catalysis of MoNi_4_ nanoalloys for Li-S batteries [55]. The calculation results implied that the MoNi_4_ nanoalloys with superficial 1.59% strain had lower lithium-ion diffusion barriers (2.878 eV) in contrast to MoNi_4_ (3.143 eV), accelerating the catalytic conversion of sulfur species.

Lithium-ion diffusion barriers can also be used to evaluate the Li deposition/stripping kinetics in Li-S batteries. Lee and co-workers reported that In_2_Se_3_ can effectively catalyze Li-S batteries and improve the reversibility of lithium deposition [100]. Acting as a dual-functional additive, In_2_Se_3_ was found to simultaneously boost the performance of cathodes and anodes for Li-S batteries. The dissolved In^3+^ and Se^2+^ reacted with polysulfides to form LiInS_2_ and LiInSe_2_, which were incorporated into the SEI and improved the plating and stripping of lithium. This can be concluded by the lithium-ion diffusion calculations. The previous report showed that the optimum pathway of lithium-ion diffusion on Li_2_S was along the (100) direction with a low barrier of 0.348 eV. In contrast, the lithium-ion diffusion barriers of LiInS_2_ and LiInSe_2_ considerably decreased to 0.286 and 0.269 eV, respectively. The smaller lithium-ion diffusion barriers confirmed more uniform lithium-ion migration through the SEI and rapid kinetics for lithium deposition.

## 6. Li_2_S Decomposition Energy Barriers

Because of the insulating nature of Li_2_S, its dissociation process during charging should overcome huge energy barriers [101]. Therefore, accelerating the catalytic Li_2_S oxidation benefits sulfur cathodes’ stable capacity and long cycle life. In this case, Zhou et al. proposed the decomposition process of Li_2_S [97]. An intact Li_2_S molecule can be decomposed into an individual lithium ion and a LiS cluster as follows:

*Li*_2_S → *LiS* + *Li*^+^ + *e*^−^_._ (13)

The decomposition process involves the dissociation of Li from the Li_2_S molecule, associated with the Li-S bond cleavage. The calculated decomposition barrier of Li_2_S can be used to evaluate the activities of catalysts towards Li_2_S oxidation. By analyzing the decomposition energy profiles of Li_2_S on various sulfides, the authors indicated that decomposition energy barriers were essentially dependent on the binding ability of the isolated lithium ions with the sulfur of sulfides. Due to the strong binding ability, sulfides caused smaller decomposition barriers than carbon since the binding of lithium ions with carbon was much weaker. This conclusion can explain why sulfides can be good catalysts for Li-S batteries.

**Figure 11:**
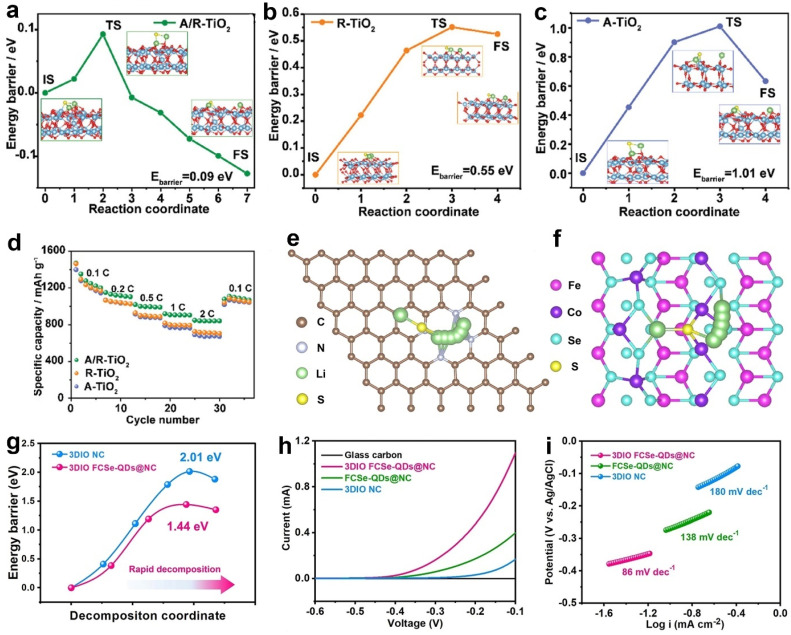
(**a**–**c**) The Li_2_S decomposition energy barriers of A/R-TiO_2_, R-TiO_2_, and A-TiO_2_. (**d**) Rate capability of different cathodes. Reproduced with permission [45]. Copyright 2023, Wiley-VCH. (**e**) Top views of the Li_2_S decomposition pathways on 3DIO NC, (**f**) 3DIO FCSe-QDs@NC, and (**g**) corresponding Li_2_S decomposition energy profiles. (**h**) LSV curves and (**i**) corresponding Tafel plots of Li_2_S oxidization. Reproduced under the terms of the CC-BY license [103]. Copyright 2023, the authors, published by Springer Nature.

Oxides have been investigated to catalyze the electrochemical conversion of Li-S batteries [80]. TiO_2_ has been confirmed to have favorable chemical adsorption for anchoring polysulfides. However, it is plagued by intrinsically low conductivity, impeding the conversion kinetics of sulfur cathodes. Wei and co-workers developed a high-performance TiO_2_ catalyst composed of a rich O-vacancy TiO_2_ anatase/rutile homojunction on carbon nanosheets (A/R-TiO_2_) [45]. The heterointerface of A/R-TiO_2_ provided effective anchoring and smooth conversion of polysulfides. It also significantly reduced the Li_2_S decomposition energy barrier. Figure 11a–c show the calculated Li_2_S decomposition energy barriers of A/R-TiO_2_, R-TiO_2_, and A-TiO_2_. A/R-TiO_2_ revealed a lower decomposition barrier of 0.09 eV compared to R-TiO_2_ (0.55 eV) and A-TiO_2_ (1.01 eV), suggesting the accelerated delithiation kinetics of Li_2_S. Jiang et al. determined the crystal facet effects of Fe_2_O_3_ on the catalytic conversion of Li-S batteries [102]. The authors developed reduced graphene oxide to load high-index faceted Fe_2_O_3_ nanocrystals, which bifunctionally catalyzed sulfur cathodes. The (1238) and (1344) facets of Fe_2_O_3_ considerably decreased the Li_2_S decomposition energy barriers in contrast to the Fe_2_O_3_ (0112) facet. This conclusion indicated that high-index facets possessed higher catalytic activities to split the S-Li bond, promoting the Li_2_S dissociation kinetics. As a result, sulfur cathodes with A/R-TiO_2_ achieved favorable capacities at various current densities (Figure 11d).

Quantum dots and single-atom catalysts have been employed in sulfur cathodes to maximize the active sites for anchoring and catalysis. Their tiny dimensions and uniform dispersibility allowed them to reduce the reaction energy barrier. For example, Huang et al. synthesized bidirectional catalysts to improve sulfur cathodes and lithium anodes [103]. The authors employed 3D inverse opal-structured N-doped carbon as the substrate to load Co-Fe selenide quantum dots (3DIO FCSe-QDs@NC). The calculation results in Figure 11e–g show that the decomposition barrier of Li_2_S on FCSe was 1.44 eV, which was smaller than that on the bare NC (2.01 eV). This result indicated an optimal dissociation process of the Li_2_S under the control of the FCSe-QDs catalytic sites. This resulted in the remarkable enhancement of the decomposition process, which was further validated by linear sweep voltammetry (LSV) tests. Figure 11h shows that 3DIO FCSe-QDs@NC displays the smallest onset voltage with the largest current response, meaning the lowest energy barrier for Li_2_S decomposition. The corresponding Tafel plots in Figure 11i also support the conclusion. 3DIO FCSe-QDs@NC showed the smallest Tafel slope among the three catalysts.

Yang et al. designed dual Ni-N_4_ and Fe-N_4_ sites co-anchored on carbon nanocages to catalyze sulfur cathodes [48]. The high ε_d_ of the Fe-N_4_ sites demonstrated an accelerated sulfur reduction reaction. Meanwhile, Li_2_S on the Ni-N_4_ sites revealed a metallic nature, leading to strong S 2p DOS near the Femi level and thus allowing small Li_2_S dissociation barriers. The calculation confirmed that the decomposition energy barrier of Li_2_S on Ni-N_4_ centers (1.20 eV) was smaller in contrast to that on Fe-N_4_ (1.35 eV). This behavior originated from the moderate anchoring ability of Li_2_S on Ni-N_4_ (−1.63 eV) compared with that on Fe-N_4_ (−2.65 eV). The moderate adsorption of Li_2_S typically resulted in favorable decomposition. Song et al. developed dual Zn-Co metal–N/O sites with combined effects on rapid catalytic kinetics for sulfur cathodes [104]. DFT calculations suggested that the Li_2_S decomposition barriers of this dual-core single-atom catalyst were lower than those of the single-core counterpart.

Other types of catalysts, such as nitrides [105], MXene [106], and heterostructures [107], have been reported to regulate Li_2_S decomposition. Ma et al. constructed a multibranched vanadium nitride (MB-VN) catalyst towards Li-S batteries with high-/low-temperature tolerance [83]. MB-VN exhibited a small Li_2_S decomposition barrier of 0.67 eV, implying a rapid Li_2_S dissociation on MB-VN. Zhang et al. reported hierarchically N-doped porous carbon incorporated F-free Ti_3_C_2_T*_x_* for Li-S batteries [106]. Ti coordinated with N presented combined effects on decreasing the Li_2_S decomposition barriers, hence accelerating the redox kinetics of sulfur cathodes. Tang and co-workers prepared Co-doped P-vacancy FeP catalysts on MXene, which considerably improved the bidirectional Li_2_S reaction processes [75]. The decomposition energy barriers of Li_2_S (0.69 eV) on the catalyst were considerably smaller in contrast to those on FeP (1.86 eV). Another MXene-based catalyst designed by Nguyen et al. has also revealed a mitigated energy barrier for Li_2_S decomposition [68].

## 7. Conclusions and Outlook

Calculations have been an essential approach to unravelling catalyst activities for Li-S batteries. Together with experiments, calculations can give comprehensive insights into the conversion mechanisms of Li-S batteries. This review summarizes the calculations on catalytic Li-S batteries. The electronic structures of catalysts, including band structures, densities of states, and charge distribution, are highly correlated with the catalytic activities. Calculating electronic structures can help us understand the intrinsic characteristics of catalysts at the atomic level. This will benefit catalyst tailoring, such as surface modification, doping, heterostructure construction, and defect engineering, aiming to enhance catalytic activities.

Binding energy calculations are critical to determining the catalysts for Li-S batteries since the mitigation of polysulfide shuttling is heavily dependent on the binding ability of catalysts. A catalyst with a weak binding energy towards sulfur species cannot anchor them into the cathode region. Therefore, the shuttle effect will persist, leading to the fast capacity decay of Li-S batteries. However, too strong binding energy is detrimental to the desorption and migration of sulfur species from the catalyst surface. Until now, there still has not been a distinct benchmark to determine if the binding energy is too high or too low. Previous reports suggest that binding energies of catalysts within the range of several eV or up to 10 eV both exhibit optimal adsorption towards sulfur species. This behavior might result from the structural nature of catalyst materials.

Note that the binding ability alone cannot fully inhibit the polysulfide shuttling. That is due to the sluggish redox kinetics of sulfur cathodes. The adsorbed polysulfides on catalysts will accumulate in the cathode region, inevitably causing their diffusion to the anode side owing to concentration gradients in the electrolyte. Therefore, catalysts that possess a strong binding ability to sulfur species and simultaneously can accelerate their conversion kinetics will successfully diminish the shuttle effect of polysulfides.

Gibbs free energy is another critical indicator to characterize the catalyst activities. The calculations of Gibbs free energy can determine which reaction step is spontaneous and which reaction is the rate-determining step during discharging. The calculated result can provide valuable information on the electrochemical reaction pathways when combined with the experimental measurements, like in situ characterization. The current calculation of the Gibbs free energy of Li-S batteries typically considers the interaction between catalysts and the representative sulfur species, i.e., S_8_ and Li_2_S*_n_* (*n* = 1, 2, 4, 6, 8). It is reasonable to some extent but not undisputable.

One reason is that the electrolyte (solvent molecules and lithium salts) strongly interacts with sulfur species. The electrolyte effects should be considered when calculating the Gibbs free energy. Another point is the inclusion of sulfur species in the calculation. The types of polysulfide intermediates are dependent on the Li-S battery system. The calculations also should consider other intermediates, like Li_2_S_3_ or polysulfide radicals. When accommodating these points, the calculation results may better reflect the conversion mechanisms of Li-S batteries.

Lithium-ion diffusion and Li_2_S decomposition energy barriers significantly affect the rate capacity of Li-S batteries since they are associated with redox kinetics. Rapid lithium-ion diffusion alleviates the accumulation of the generated polysulfides at the cathode surface during cycling, thus diminishing their shuttling in the electrolyte. However, as lithium-ion diffusion is related to polysulfide diffusion, too fast diffusion of lithium ions is not always beneficial. It will result in the weak adsorption of polysulfides at the surface of catalysts. A trade-off between lithium-ion diffusion and polysulfide adsorption should be considered when designing effective catalysts. Li_2_S decomposition involves the charging process in Li-S batteries. Understanding the bidirectional catalytic mechanisms of catalysts on both discharging and charging is critical for stable Li-S batteries.

The applications of current theoretical calculations on Li-S batteries have achieved considerable success. DFT calculations present great advantages in computational accuracy without the extra increase in computing time. The electronic structures of catalyst materials and conversion mechanisms of sulfur cathodes are well elucidated and predicted. However, the calculation results are not always consistent with the experimental measurements. This deviation mainly originates from the number of particles used for calculation. DFT methods typically choose a finite number of particles to calculate and analyze the physical or chemical properties of catalyst materials. The number of calculated particles is far less than that of the actual materials. Therefore, the calculation results generally reflect the localized and static characteristics of catalysts. To overcome the drawbacks, the DFT calculation must include many more particles and proper configurations of catalyst materials. However, the improved calculation accuracy might be achieved at the expense of computing time. A fundamental trade-off between calculation accuracy and computing time should be considered.

Based on the calculation studies associated with experimental analyses, the most promising catalyst materials for Li-S batteries should possess the following advantages: (1) Favorable conductivity for rapid charge transport that can be analyzed with electronic structure calculations and lithium-ion diffusion barriers; (2) moderate anchoring towards sulfur species, which is validated by the binding energy calculations; and (3) effective active sites for catalyzing the conversion of sulfur species. The conversion step of sulfur species with a lower calculated Gibbs free energy indicates the thermodynamically more favorable pathway. In addition, catalyst materials with high structural stability and low cost are desirable for large-scale applications. Nowadays, finding catalyst materials for Li-S batteries that can meet all the criteria is challenging. Still, carbon-based catalysts and metal sulfides are expected to show considerable potential for enhancing the performance of Li-S batteries.

As discussed in this review, although tremendous efforts with theoretical calculations have been made to unravel the catalysis in Li-S batteries, a deep understanding of the catalytic mechanisms of the multistep conversion reactions is still limited. Therefore, there is much work to be conducted regarding theoretical calculations which explore efficient catalysts for Li-S batteries. First, the complicated disproportionation and neutralization of polysulfides in the electrolyte pose a significant challenge for analyzing the sulfur species quantitatively. Understanding the specific conversion process of various sulfur species can provide the foundation for revealing the catalytic mechanisms of Li-S batteries. This is difficult for experimental approaches, and theoretical calculations may be crucial. In addition, predicting and screening efficient and stable catalysts for Li-S batteries needs tremendous effort. Developing advanced calculation approaches can facilitate the discovery of catalyst materials. Machine learning associated with high-throughput screening strategies is a promising method for exploring ideal catalysts. The data-driven calculations based on theoretical models show great potential for efficiently predicting the properties of catalyst materials, like adsorption configurations and adsorption energies. More importantly, the advanced calculation methods remove the obstacle facing experimental technologies, which seek catalyst materials through trial and error.

Calculations have considerably promoted the understanding of the electrocatalytic reactions of Li-S batteries. Many new and special calculation methods have been developed and employed to unravel the complicated Li-S electrochemistry. Together with experimental characterization, these calculations will immensely accelerate the practical applications of Li-S batteries.

## Data Availability

Not applicable.
